# The complete mitochondrial genome of *Tylonycteris fulvida* (Chiroptera: Vespertilionidae) in South China

**DOI:** 10.1080/23802359.2021.1993103

**Published:** 2021-10-23

**Authors:** Weijian Guo, Xiaoling Liang, Yi Wu, Wenhua Yu

**Affiliations:** a Jiangsu Key Laboratory for Biodiversity and Biotechnology, College of Life Sciences, Nanjing Normal University, Nanjing, China; b Key Laboratory of Conservation and Application in Biodiversity of South China, School of Life Sciences, Guangzhou University, Guangzhou, China

**Keywords:** Chiroptera, mitochondrial genome, *de novo* assembly, *Tylonycteris fulvida*

## Abstract

The complete mitochondrial genome of *Tylonycteris fulvida* (Peters, 1872) was obtained using high-throughput sequencing technology. The genome is a circular molecule of 16,621 bp length, containing 13 protein-coding genes, 2 *r*RNA genes, 22 *t*RNA genes, and a control region. A phylogenetic tree of 13 protein-coding genes was constructed using IQ-TREE. Our result suggests that *T. fulvida* cluster within Chiroptera and Fereuungulata. The complete mitochondrial genome sequence of *T. fulvida* will be helpful for future taxonomic and phylogenetic studies on Chiroptera.

The lesser bamboo bat (*Tylonycteris fulvida*) is belonging to Laurasiatheria, Chiroptera, Vespertilionidae, which are the first family including most speciose in bats (Amador et al. 2018). The previous phylogenetic study suggested two popular hypotheses among Laurasiatheria, i.e. the ‘Fereuungulata’ and the ‘Pegasoferae’ hypothesis (Murphy et al. 2007; dos Reis et al. 2012; Foley et al. 2016; Esselstyn et al. 2017). These hypotheses are related to which order (Chiroptera or Cetartiodactyla) is the seconding-branching laurasiatherian lineage. In this study, we generated a complete mitogenome of *T*. *fulvida* (GenkBank accession: MZ457524) from China which maybe contributes to our understanding of the phylogenetic relationship within Laurasiatheria.

An adult female of *T*. *fulvida* (Voucher No. GZHU17245) was collected Guangdong Province, China (23°17′25.9″N, 113°24′35.8″E). The liver tissue was kept in 95% ethanol at −20 °C laboratory freezer in Guangzhou University (CONTACT: Wenhua Yu, email: wenhua_yu@gzhu.edu.cn). Complete genomic DNA was extracted from MiniBEST Universal Genomic DNA Extraction Kit (TAKARA, Dalian) and was sequenced using MGISEQ-2000RS with a PE150 protocol. Following the MitoZ tutorial (Meng et al. 2019), a total of 5 G base pairs (bp) were obtained, then a complete mitochondrial genome was further generated and automatically annotated.

The complete mitogenome of *T*. *fulvida* is 16,621 bp long with a base composition of 12.75% G, 34.48% A, 30.37% T, and 22.40% C. It encoded 37 genes including 13 protein-coding genes, 22 *t*RNA genes, 2 *r*RNA genes, and a control region (*D*-loop). All these genes were encoded on the heavy strand, except for the *ND6* protein-coding gene and eight tRNA genes (*t*RNA^Gln^, *t*RNA^Ala^, *t*RNA^Asn^, *t*RNA^Cys^, *t*RNA^Tyr^, *t*RNA^Ser^, *t*RNA^Glu^, and *t*RNA^Pro^) which were located on light strand. The total length of protein-coding genes was 11,436 bp, occupying 68.80% of the total length. All protein initiation codons are ATG, except for *ND2*, *ND5* with ATA, and *ND3* with ATT. Nine protein-coding genes (*ND1*, *COX1*, *COX2*, *ATP8*, *ATP6*, *ND4L*, *ND5*, *ND6*, Cyt *b*) terminate with the stop codon TAA, while the *ND2* and *ND3* end with TAG. Besides, incomplete stop codon (T-- or TA-) was observed in *ND4* and *COX3*, respectively.

To verify the phylogenetic hypothesis, the 13 protein-coding genes of 19 laurasiatherian species were used to reconstruct the phylogenetic tree that was rooted by *Condylura cristata* and *Sorex araneus* (Figure 1). All the sequences were aligned using MAFFT (Katoh and Standley 2013) and subsequently cleaned using Gblocks (Castresana 2000). Next, the best model for each protein-coding gene was selected using ModelFinder (Kalyaanamoorthy et al. 2017) as implemented in IQ-TREE v. 1.6.12 (Nguyen et al. 2015). Following the model selection, the best ML (maximum likelihood) tree was estimated with 1000 ultrafast bootstraps (UFboot) (option: -bb 1000) (Hoang et al. 2018) and the SH-like approximate likelihood ratio test (SH-aLRT) (option: -alrt 1000) (Guindon et al. 2010) using IQ-TREE. The ML tree showed that the Chiroptera was the seconding-branching laurasiatherian lineage with 100% bootstrap support which supported the ‘Fereuungulata’ hypothesis. Mitochondrial genome of *T*. *fulvida* could benefit future phylogenetic and evolutionary studies on Laurasiatheria and Chiroptera.

**Figure 1.**
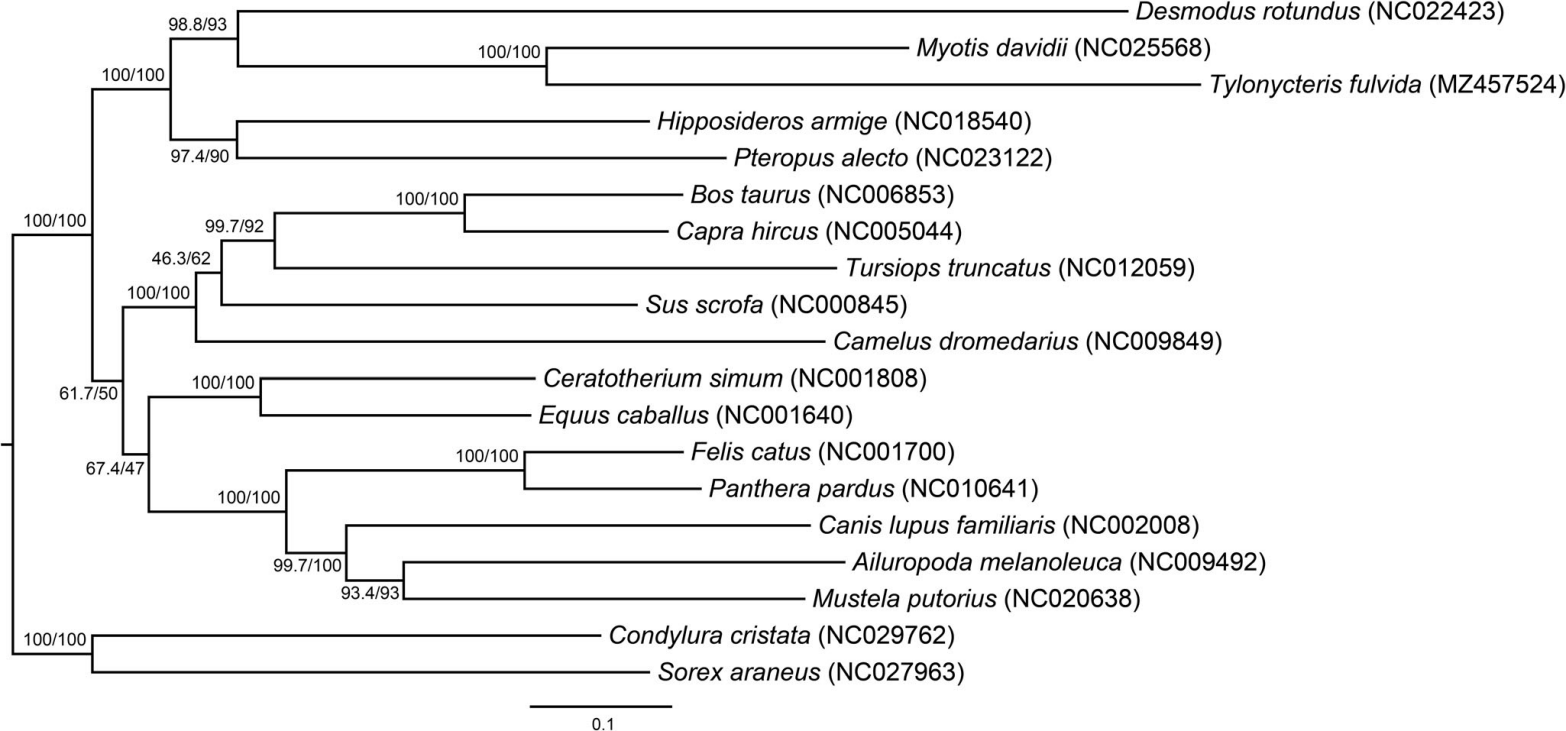
Phylogenetic tree based on 13 protein-coding genes of 19 laurasiatherian species reconstructed using the ML methods. Numbers above each branch represent bootstrap and branch supports. GenBank accession number of each species is shown in parentheses.

## Data Availability

The data that support the findings of this study are openly available in NCBI at https://www.ncbi.nlm.nih.gov/nuccore/ MZ457524, reference number MZ457524. The associated BioProject, SRA and Bio-Sample numbers are PRJNA741550, SRR15195148 and SAMN19884333, respectively.
